# Isoferulic acid prevents methylglyoxal-induced protein glycation and DNA damage by free radical scavenging activity

**DOI:** 10.1186/s12906-015-0874-2

**Published:** 2015-10-05

**Authors:** Aramsri Meeprom, Weerachat Sompong, Tanyawan Suantawee, Thavaree Thilavech, Catherine B. Chan, Sirichai Adisakwattana

**Affiliations:** Program in Clinical Biochemistry and Molecular Medicine, Department of Clinical Chemistry, Faculty of Allied Health Sciences, Chulalongkorn University, Bangkok, 10330 Thailand; Program in Biomedical Sciences, Graduate School, Chulalongkorn University, Bangkok, 10330 Thailand; Research Group of Herbal Medicine for Prevention and Therapeutic of Metabolic diseases, Department of Nutrition and Dietetics, Faculty of Allied Health Sciences, Chulalongkorn University, Bangkok, 10330 Thailand; Departments of Physiology and Agricultural, Food and Nutritional Sciences, University of Alberta, Edmonton, Alberta, T6G 2R3 Canada; Department of Nutrition and Dietetics, Faculty of Allied Health Sciences, Chulalongkorn University, Bangkok, 10330 Thailand

**Keywords:** Isoferulic acid, Methylglyoxal, Advanced glycation end products, DNA damage, Oxidation

## Abstract

**Background:**

Isoferulic acid (IFA), a naturally occurring cinnamic acid derivative, is a main active ingredient of the rhizoma of *Cimicifuga dahurica.* It has been shown various pharmacological activities. The aim of the study was to investigate the effect of IFA against MG-induced protein glycation and oxidative DNA damage. Free radical scavenging activity and the MGO-trapping abilities of IFA were also investigated.

**Methods:**

The fluorescent MG-derived AGEs and non-fluorescent N^ε^-(carboxymethyl) lysine (N^ε^-CML) was measured using a spectrofluorometer and an enzyme linked immunosorbant assay (ELISA). Protein carbonyl content was used to detect protein oxidation. Gel electrophoresis was used to determine DNA damage. Superoxide anion radicals and hydroxyl radicals were determined using cytochrome *c* reduction assay and thiobarbituric acid reactive 2-deoxy-D-ribose oxidation products, respectively. The MG-trapping capacity was performed by HPLC.

**Results:**

IFA (1.25–5 mM) inhibited the formation of fluorescent MG-derived AGEs, and N^ε^-CML, and protein carbonyl in bovine serum albumin. In addition, IFA (0.1–1 mM) also prevented MG/lysine-mediated oxidative DNA damage in the presence and absence of copper ion. The protective ability of IFA was directly correlated to inhibition of hydroxyl and superoxide anion radical generation during the reaction of MG and lysine. Most notably, IFA had no the directly trapping ability to MG.

**Conclusions:**

The present results highlighted that free radical scavenging activity, but not the MG-trapping ability, is the mechanism of IFA for preventing MG-induced protein glycation and DNA damage.

## Background

Methylglyoxal (MG), a highly reactive α-oxalaldehyde metabolite, is formed endogenously during glucose, protein and fatty acid metabolism. Other sources of MG, which are formed during industrial processing and long-term storage, are in sugar-containing foods and beverages, such as bread, coffee, honey, wine, and beer [1]. Increased MG levels are possible causal factors for development and progression of diabetes and its complications [2]. MG readily reacts with lysine and arginine residues of protein to produce non-enzymatic protein glycation and subsequent formation of advanced glycation end-products (AGEs), crosslinks like methylglyoxal-lysine dimers, and N^ε^-(carboxymethyl) lysine (N^ε^-CML) [3]. The consequences of these reactions alter the characteristics of proteins and their physiochemical and biochemical properties. In vitro experiments have recently shown that reactive oxygen species (ROS) are also generated during the glycation reaction of protein with MG. This results in depletion of thiol-containing protein and an increase in protein carbonyl formation [4]. Besides direct glycation damage to protein, MG reacting with lysine may contribute to oxidative DNA damage, strand breakage and cell apoptosis [5, 6]. Moreover, Cu^2+^ enhances MG-lysine mediated DNA damage, participating in a Fenton’s-type reaction to produce hydroxyl radicals [7]. ROS-induced oxidative DNA damage has been causally associated with the mechanism of mutagenesis [8]. In this regards, application of AGE inhibitors has emerged as a new strategy to reduce the occurrence of AGE-associated diseases. Recent attention has focused on identification of AGE inhibitors from phytochemical compounds that act as antioxidants, chelate metal ions, or directly trap MG [9].

Cinnamic acid and its derivatives are widely distributed among fruits and vegetables in the human diet. They exert many biological effects such as anti-inflammatory [10], anti-oxidation [11], and anti-hyperglycemic activities [12]. Isoferulic acid (IFA), a naturally occurring cinnamic acid derivative, is a main active ingredient of the rhizoma of *Cimicifuga dahurica* [13], which targets multiple pathways associated with antihyperglycemic activity. In vitro and in vivo studies demonstrate that IFA has a plasma glucose-lowering effect in streptozotocin-induced diabetic rats [14]. The mechanism of its action involves activation of α_1_-adrenoceptors to enhance the secretion of β-endorphin, which can stimulate the opioid μ-receptors [15, 16]. The action leads to increased glucose utilization and reduced hepatic gluconeogenesis. In addition, IFA is the most inhibitor against intestinal α-glucosidase among 11 cinnamic acid derivatives [17]. Most interestingly, IFA acts as an anti-glycating agent against fructose- and glucose-induced protein glycation and oxidation-dependent damage to protein [18]. However, no information exists on the abilities of IFA to inhibit MG-induced protein glycation and DNA damage.

The aim of the present work was to investigate the inhibitory effect of IFA on MG-induced protein glycation and oxidative damage using bovine serum albumin (BSA). Moreover, a glycation model system consisting of lysine and MG together with Cu^2+^ was created to investigate the ability of IFA to prevent oxidative DNA damage. Furthermore, IFA was evaluated for its free radical scavenging activity in the model of lysine/MG and the capacity in direct trapping of MG using HPLC.

## Methods

### Chemicals and reagents

Methylglyoxal (40 % in water), isoferulic acid (IFA, 3-hydroxy-4-methoxycinnamic acid), bovine serum albumin (BSA, fraction V), aminoguanidine, 5,5’-dithiobis (2-nitrobenzoic acid) (DTNB), 2-deoxy-D-ribose, 2-methylquinoxaline, 5-methylquinoxaline, *o*-phenylenediamine, thiobarbituric acid and cupric sulfate (CuSO_4_) were purchased from Sigma-Aldrich (St.Louis, MO, USA). L-lysine, 2,4-dinitrophenyl hydrazine (DNPH) and guanidine hydrochloride were obtained from Himedia (Mumbai, India), Ajax Finechem (Taren Point, Australia) and Fluka (Steinheim, Germany), respectively. OxiSelect™ N^ε^-(carboxymethyl) lysine (CML) ELISA kit was acquired from Cell Biolabs (San Diego, CA, USA). QIAprep Spin Miniprep kit was obtained from Qiagen (Venlo, Netherlands) and cytochrome *c* was purchased from Affymetrix (Santa Clara, CA, USA). All other chemicals used were of analytical grade.

### Glycation of bovine serum albumin (BSA) by methylglyoxal

The glycated BSA formation assay was modified according to a previously published method [19]. The reaction mixtures (1 mL per reaction) containing 460 μL of methylglyoxal (MG, at final concentration of 1 mM), 500 μL of 20 mg/mL BSA (final concentration: 10 mg/mL) in 0.1 M phosphate buffered saline (PBS, pH 7.4) and 40 μL of IFA at various concentrations (final concentrations: 1.25, 2.5 and 5 mM) or aminoguanidine (AG, final concentration: 1.25 mM) were incubated at 37 °C for 2 weeks. All glycated samples were taken for analysis of fluorescent MG-derived AGEs, non-fluorescent N^ε^-CML, and carbonyl content.

### Measurement of fluorescent MG-derived AGEs

The fluorescent intensity was measured weekly to assess MG-derived AGEs by using the excitation and emission wavelengths at 355 and 460 nm, respectively. The inhibitory effect of IFA on fluorescent MG-derived AGEs was calculated as percentage inhibition following formula below:

$$ \begin{array}{l}\%\mathrm{Inhibition} = \frac{\left({\mathrm{F}}_{\mathrm{C}}-{\mathrm{F}}_{\mathrm{C}\mathrm{B}}\right)-\left({\mathrm{F}}_{\mathrm{S}}-{\mathrm{F}}_{\mathrm{S}\mathrm{B}}\right)}{{\mathrm{F}}_{\mathrm{C}} - {\mathrm{F}}_{\mathrm{C}\mathrm{B}}}\kern4.5em \times 100\kern3em \\ {}\kern2.50em \end{array} $$

Where F_C_ and F_CB_ were the fluorescent intensity of control with MG and blank of control without MG, F_S_ and F_SB_ were the fluorescent intensity of IFA with MG and blank of IFA without MG.

### Measurement of non-fluorescent N^ε^-CML

Non-fluorescent N^ε^-(carboxymethyl) lysine (N^ε^-CML) was measured using an enzyme linked immunosorbant assay (ELISA) kit according to the manufacturer’s instruction. The absorbance of samples was measured immediately at 450 nm and compared with the absorbance of CML-BSA standard provided in the assay kit.

### Determination of protein carbonyl content

The carbonyl content in glycated BSA was determined according to a previously published method with slight modifications [6]. Briefly, 10 mM DNPH in 2.5 M HCl (400 μL) was added to 100 μL of glycated samples and incubated for 1 h in the dark. Thereafter, 500 μL of 20 % (w/v) trichloroacetic acid (TCA) was added to precipitate protein for 5 min on ice and then centrifuged at 10,000 *g* for 10 min at 4 °C. The protein pellet was washed with 1:1 (v/v) ethanol/ethyl acetate mixture three times and resuspended in 250 μL of 6 M guanidine hydrochloride. The absorbance was read at 370 nm. The carbonyl content of each sample was calculated based on the molar extinction coefficient for DNPH (*ε* = 22,000 M^−1^ cm^−1^) and final results were expressed as nmol carbonyl/mg protein.

### Plasmid extraction

The pUC19 plasmid was purified from competent *Escherichia coli* by using QIAprep Spin Miniprep kit according to the manufacturer’s protocol and measured DNA concentration using the NanoDrop-1000 spectrophotometer (Thermo Scientific, MA, USA). The plasmid was kept at −20 °C until use.

### DNA strand breakage

The assessment of DNA strand breakage was performed according to a previous publication with minor modifications [7]. Extracted plasmid containing 0.25 μg DNA was incubated with 2 μL of 250 mM lysine (final concentration: 50 mM), 2 μL of 250 mM MG (final concentration: 50 mM), and 2 μL of IFA at various concentrations (final concentration: 0.1, 0.25, 0.5 and 1 mM) with or without 1 μL of 3 mM CuSO_4_ (final concentration: 300 μM) in a total volume of 10 μL at 37 °C for 3 h. The reaction was stopped at −20 °C for 90 min before mixing with DNA loading dye and loading onto 0.8 % agarose gel in Tris-borate-EDTA (TBE) buffer. The gel was electrophoresed at 80 V for 60 min, stained with ethidium bromide and photographed by Gel Doc imager (Syngene, UK). Plasmid DNA fragments detected on agarose gel consisted of supercoiled form (SC) and open circular form (OC). The intensity of each band was analyzed using GeneTools software (Syngene, UK). The results were expressed as the percentage of opened circular (% OC) calculated using the following formula before subtracting by % OC of untreated-DNA control.

$$ \%\mathrm{O}\mathrm{C}\kern.5em =\kern.5em \frac{\mathrm{Intensity}\kern0.5em \mathrm{of}\kern0.5em \mathrm{O}\mathrm{C}}{\mathrm{Intensity}\kern0.5em \mathrm{of}\left(\mathrm{O}\mathrm{C} + \mathrm{S}\mathrm{C}\right)} \times 100 $$

### Determination of superoxide anion

Superoxide anion was determined by using cytochrome *c* reduction assay with minor modifications [7]. The reaction mixtures with equal volumes (200 μL) of 50 mM lysine and 50 mM MG (final concentration: 10 mM) with or without 100 μL of IFA (final concentrations: 0.1, 0.25, 0.5 and 1 mM) were adjusted to a total volume of 900 μL before adding 100 μL 300 μM cytochrome *c* (final concentration: 30 μM) and monitoring the production of superoxide anion by measuring reduced cytochrome *c* at a wavelength of 550 nm every 10 min until 180 min. The concentration of reduced cytochrome *c* at each time point was calculated using its molar extinction coefficient (27,700 M^−1^cm^−1^) and then subtracting baseline (at 0 min). The results were expressed as nmol/mL.

### Determination of hydroxyl radical

Hydroxyl radical was determined by measuring thiobarbituric acid reactive 2-deoxy-D-ribose oxidation products (TBARS) according to a previously published method with minor modifications [7]. The reaction contained equal volumes (20 μL) of 50 mM lysine, 50 mM MG (final concentration: 10 mM) and 100 mM 2-deoxy-D-ribose (final concentration: 20 mM) with or without 20 μL of IFA (final concentrations: 0.1, 0.25, 0.5 and 1 mM). The volume was adjusted to 100 μL using 10 mM PBS before incubating at 37 °C. After 3 h of incubation, the mixture was added to an equal volume of 10 mM PBS (100 μL), 2.8 % (w/v) TCA and 1 % (w/v) thiobarbituric acid (TBA), followed by heating at 100 °C for 10 min, then cooling to room temperature. The degradation of 2-deoxy-D-ribose was measured using a spectrophotometer at a wavelength of 532 nm. The concentration of TBARS was calculated from malondialdehyde (MDA) standard and the results were expressed as nmol/mL.

### Determination of the MG-trapping capacity by HPLC

The MG-trapping capacity was done according to a previously published method with minor modifications [20]. A mixture of MG (1 mM) with IFA (1.25, 2.5 and 5 mM) or AG (1 mM) in phosphate buffer solution (pH 7.4) at 37 °C was incubated for 1 h and 24 h. Quantification of MG was based on the determination of its derivative compound, 2-methylquinoxaline (2-MQ) using HPLC with 5-methylquinoxaline (5-MQ) as the internal standard. The solution containing 20 mM *o*-phenylenediamine (*o*-PDA) (100 μL) and 5 mM 5-MQ (100 μL) was added into the sample vials immediately after MG/compound incubation. MG derivatization took place at room temperature. After 30 min, the samples were filtered prior to HPLC analysis. The remaining non-derivatized MG in samples was quantified using HPLC (Shimadzu Corp., Kyoto, Japan) equipped with a LC-10 AD pump, SPD-10A UV–vis detector and LC-Solution software. A C_18_ (Inertsil ODS 3 V) column (250 × 4.6 mm i.d.; 5-μm particle size) was used for 2-MQ analysis. The column was maintained at room temperature. The mobile phase for the HPLC system consisted of HPLC grade water (solvent A) and methanol (solvent B) with a constant flow rate set at 1.2 mL/min. In brief, aliquots of 10 μL were subjected to HPLC analysis. An isocratic program was performed with 70 % solvent B and 11-min running time per sample. The 2- and 5-MQ was monitored at 315 nm. Peak integrality ratios of 2-MQ to 5-MQ were used for quantitative analysis. The amount of MG was calculated by using the standard curve of 2-MQ/5-MQ.The percentage of MG reduction was calculated using the equation below.

$$ \begin{array}{l}\ \%\ \mathrm{Reduction} = \kern0.75em \frac{\mathrm{Amount}\ \mathrm{of}\ \left(\mathrm{M}\mathrm{G}\ \mathrm{in}\ \mathrm{control} - \mathrm{M}\mathrm{G}\ \mathrm{in}\ \mathrm{test}\ \mathrm{compound}\right)}{\ \mathrm{Amount}\kern0.5em \mathrm{of}\kern0.5em \mathrm{M}\mathrm{G}\kern0.5em \mathrm{in}\mathrm{control}\kern3em } \times 100\hfill \\ {}\hfill \end{array} $$

### Statistical analysis

All data are presented as means ± SEM. In the experiment of MG-derived AGEs, two-way ANOVA was evaluated for the significant differences among groups. Other experiments were analyzed the significant differences by one-way ANOVA. Duncan’s post-hoc test was used to examine differences among groups. A *p*-value < 0.05 was considered statistically significant.

## Results

### Effect of IFA on the formation of fluorescent MG-derived AGEs and protein oxidation

**Fig. 1:**
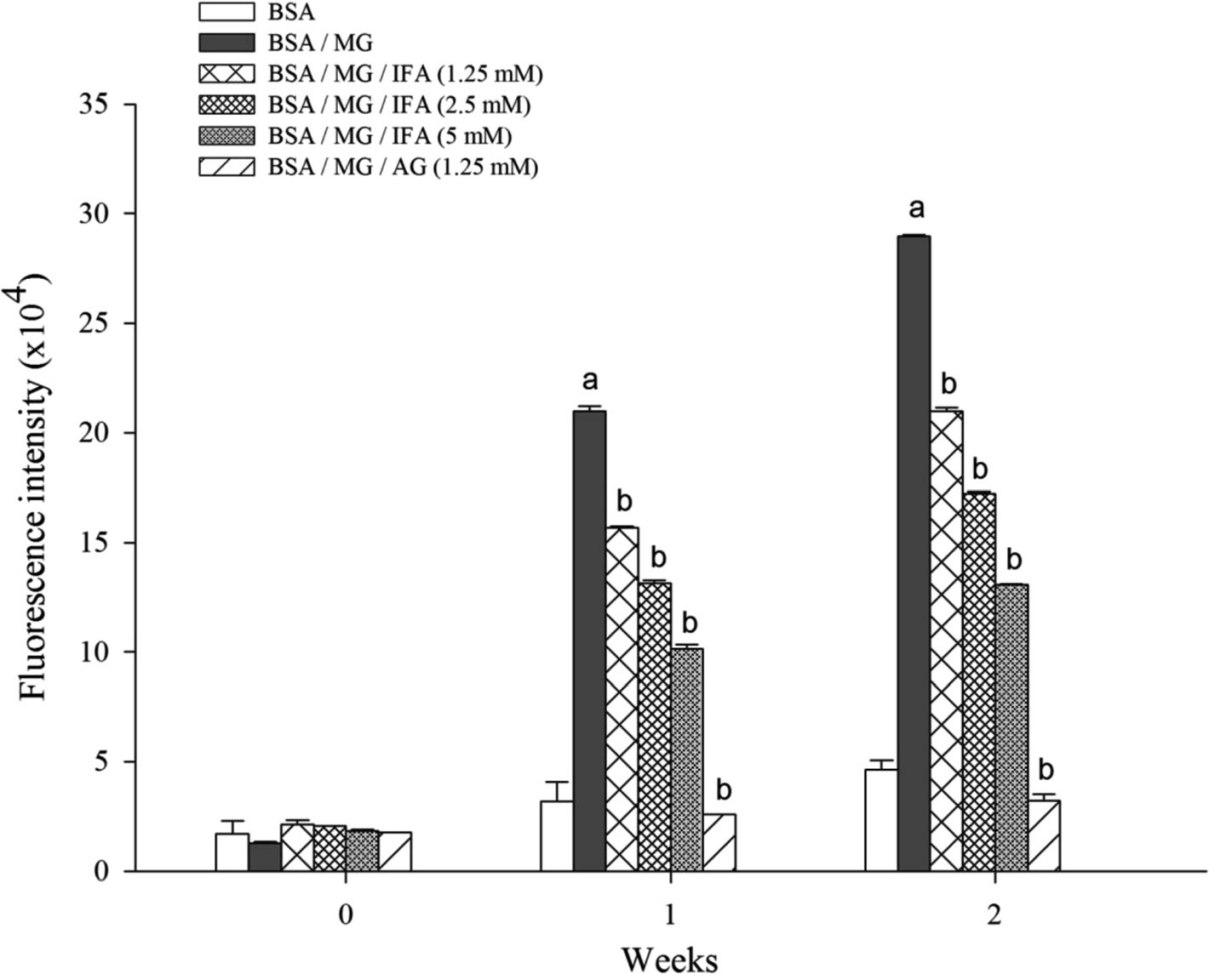
Effect of IFA on fluorescent MG-derived AGEs at week 1 and 2. The excitation and emission wavelengths were at 355 and 460 nm, respectively. The results are presented as mean ± SEM (*n* = 3). ^a^ *p* <0.05 when compared to BSA at the same week; ^b^ *p* <0.05 when compared to BSA/MG at the same week

Figure 1 depicts the fluorescence intensity of BSA incubated with MG and IFA for up to 2 weeks. A 16- and 20-fold increase of fluorescence intensity, respectively, was observed for BSA incubated with MG after 1 and 2 weeks. When IFA (1.25–5 mM) was incubated with MG, the fluorescence intensity significantly decreased. The inhibition by IFA (1.25–5 mM) ranged from 25.4 to 51.6 % at week 1 and from 27.5 to 54.9 % at week 2. In addition, AG (1.25 mM) completely inhibited fluorescent MG-derived AGEs. The level of N^ε^-CML and the concentration of protein carbonyl were determined at the end of 2-weeks incubation (Table 1). The results demonstrated that MG caused a 2-fold increase of N^ε^-CML formation as compared to BSA controls. IFA (1.25–5 mM) markedly reduced the formation of N^ε^-CML (29.9–41.3 %, *p* <0.05 for all concentrations). A similar effect was observed with 1.25 mM AG (37.0 % inhibition). The protein carbonyl concentration in BSA incubated with MG was 13.5-fold higher than that of BSA. AG suppressed MG-induced carbonylation in BSA (78.3 %) whereas IFA (5 mM) significantly (*p* <0.05) reduced the elevated carbonyl content by a maximum of 52.3 %.

**Table 1:** Effect of IFA on the formation of non-fluorescent N^ε^-CML and carbonyl content in BSA incubated with MG at week 2

Experiments	N^ε^-CML	Carbonyl content
	(ng/mL)	(nmol/mg protain)
BSA	2.97 ± 0.21	0.14 ± 0.02
BSA / MG	5.75 ± 0.50^a^	1.89 ± 0.11^a^
BSA / MG / IFA (1.25 mM)	4.03 ± 0.44^b^	1.34 ± 0.40^b^
BSA / MG / IFA (2.5 mM)	3.93 ± 0.27^b^	0.97 ± 0.08^b^
BSA / MG / IFA (5 mM)	3.37 ± 0.44^b^	0.90 ± 0.11^b^
BSA / MG / AG (1.25 mM)	3.62 ± 0.40^b^	0.41 ± 0.08^b^

The results are presented as mean ± SEM (*n* = 3). ^a^ *p* <0.05 when compared to BSA; ^b^ *p* <0.05 when compared to BSA/MG

### Effect of IFA on MG/lysine-induced DNA strand breakage

**Fig. 2:**
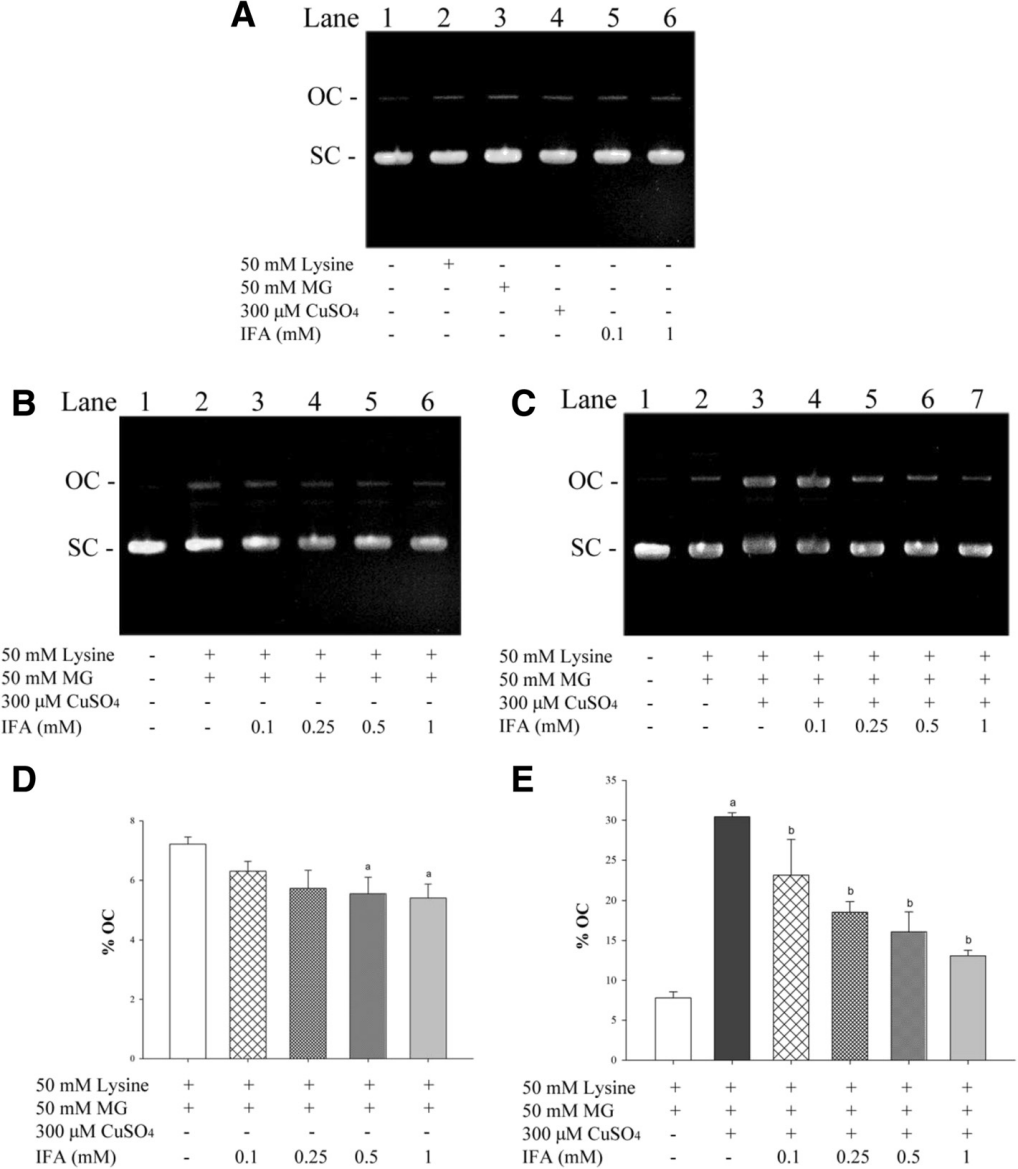
Effect of IFA on lysine/MG-induced DNA strand breakage in the absence or presence of Cu^2+^. Gel images express the intensity of open circular (OC) and supercoiled (SC) forms of plasmid DNA after pUC19 (0.25 μg) was incubated with the following treatments: (**a**) 50 mM lysine, 50 mM MG, 300 μM CuSO_4_ or IFA (0.1 and 1 mM). (**b**) Lysine and MG in the absence or presence of various IFA concentrations (0.1–1 mM) without addition of CuSO_4_. (**c**) Lysine and MG in the absence or presence of various IFA concentrations (0.1–1 mM) co-incubating with CuSO_4_. The percentage of OC (% OC, see calculation method in Methods section) in each treatment was subtracted by % OC of untreated DNA before expressing in (**d**) and (**e**) which represents data from gel (**b**) and (**c**), respectively. The results are presented as mean ± SEM (*n* = 3). ^a^ *p* <0.05 when compared to lysine/MG; ^b^ *p* <0.05 when compared to lysine/MG/Cu^2+^

The effect of IFA on preventing DNA strand breakage is presented in Fig. 2. Untreated plasmid and damaged plasmid DNA were detected as major bands of supercoiled (SC) and open circular (OC) forms, respectively. Control experiments in which plasmid DNA was incubated with lysine or MG or Cu^2+^ or IFA (0.1 and 1 mM) alone had no significant effect on detection of the OC form (Fig. 2a) whereas incubation of DNA together with lysine and MG markedly induced DNA stand breakage with 2-fold increase in the intensity of the OC band (Fig. 2b). IFA significantly reduced DNA damage at 0.5 mM (23.0 %) and 1 mM (24.9 %) whereas IFA at 0.25 mM and 0.1 mM did not have a significant effect (Fig. 2d). The cleavage of lysine/MG-treated DNA was enhanced by addition of Cu^2+^ as shown by an increase in the intensity of OC and a decrease in the intensity of SC (Fig. 2c). In the presence of Cu^2+^, DNA strand breakage was inhibited by IFA (0.1–1 mM) with percent inhibition values ranging from 24.0 to 57.0 % (Fig. 2e).

### Effect of IFA on MG/lysine-induced production of superoxide anion and hydroxyl radical

**Fig. 3:**
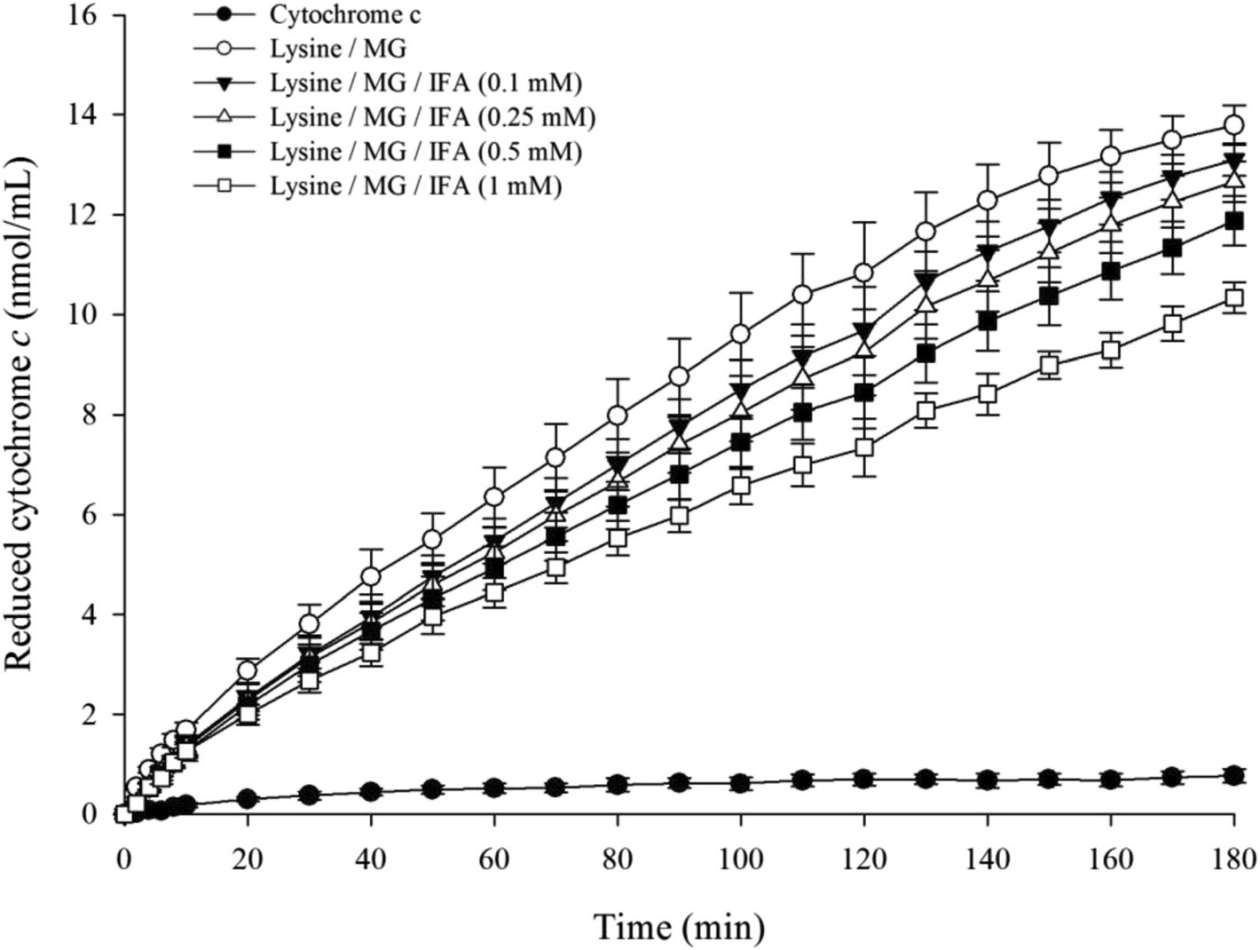
Effect of IFA on the production of superoxide anion in lysine/MG-induced glycation as measured by cytochrome *c* reduction within 180 min. The results are presented as mean ± SEM (*n* = 3)

To monitor the generation of superoxide anion induced by the reaction between MG and lysine, the reduction of cytochrome *c* was used as an indicator. Figure 3 represents a time-dependent increase of the reduced form of cytochrome *c,* corresponding to increased superoxide anion production during 180 min of incubation. Superoxide anion produced by the interaction of lysine with MG created 13.8 nmol/mL reduced cytochrome *c* at 180 min (Table 2). The generation of superoxide anion was suppressed by IFA after 10 min of incubation. At the end of incubation, IFA (0.1–1 mM) inhibited lysine/MG-induced superoxide anion production (5.0–25.0 %). Table 2 shows the TBARS concentration in the MG-lysine system indicating the generation of hydroxyl radicals. Similar to the effect on superoxide anion formation, IFA had the ability to reduce the generation of hydroxyl radical. The percentage inhibition of hydroxyl radical generation by IFA (0.1–1 mM) ranged from 26.1 to 44.6 %.

**Table 2:** Effect of IFA on the production of superoxide anion and hydroxyl radical in lysine/MG glycation at the time point of 180 min

Experiments	Reduced cytochrome *c*	TBARS
	(nmol/mL)	(nmol/mL)
Lysine / MG	13.79 ± 0.40	0.96 ± 0.03
Lysine / MG / IFA (0.1 mM)	13.10 ± 0.33	0.71 ± 0.03^a^
Lysine / MG / IFA (0.25 mM)	12.66 ± 0.41	0.58 ± 0.03^a^
Lysine / MG / IFA (0.5 mM)	11.88 ± 0.49^a^	0.56 ± 0.03^a^
Lysine / MG / IFA (1 mM)	10.34 ± 0.30^a^	0.53 ± 0.04^a^

The results are presented as mean ± SEM (*n* = 3). ^a^ *p* <0.05 when compared to lysine/MG

### Effect of IFA on MG-trapping capacity

**Fig. 4:**
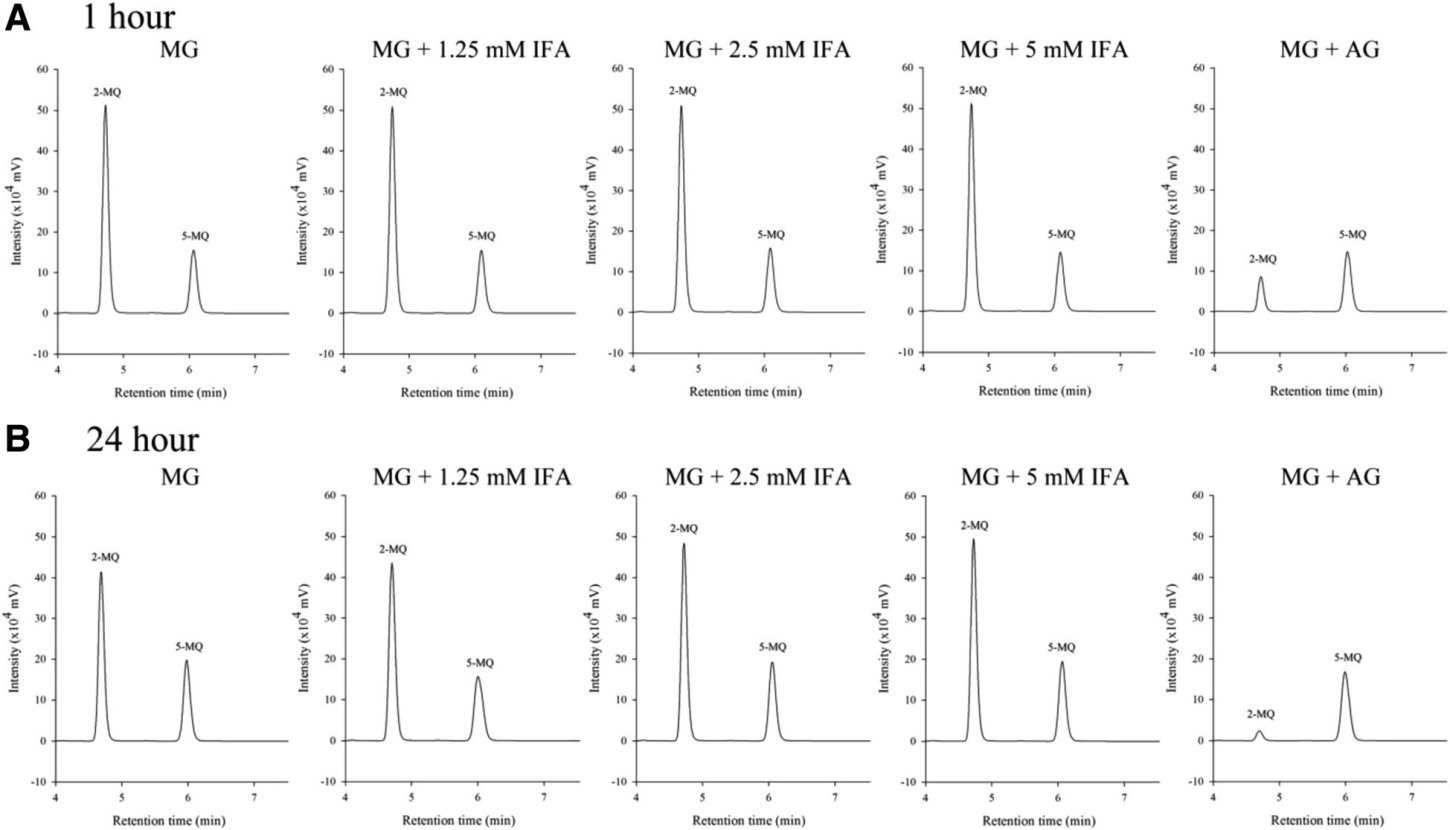
The HPLC chromatogram of MG after reaction with IFA or AG at 1 h (**a**) and 24 h (**b**). MG was detected as 2-methylquinoxaline (2-MQ) after derivatization using *o*-phenylenediamine at 315 nm. 5-methylquinoxaline (5-MQ) was used as the internal standard

An evaluation of direct MG-trapping capacity was carried out in order to investigate whether IFA could directly scavenge MG. Fig. 4a and b demonstrate the direct MG-trapping ability of IFA and AG after 1 h and 24 h of incubation. The level of 2-MQ, a product from the reaction of MG and *o*-PDA, represented free MG remaining from the trapping reaction. The results showed that AG exhibited MG-trapping ability with the values of 82.9 % at 1 h and 93.5 % at 24 h. However, IFA (1.25–5 mM) had no MG-trapping ability after 1 h and 24 h of incubation.

## Discussion

The formation of AGEs is classified into three stages: early, intermediate, and late [21]. The reaction between protein and reducing sugars (glucose and fructose) results in Schiff base formation followed by rearrangement to an Amadori product, referred to as the initial stage of glycation. In the intermediate stage, reactive dicarbonyls, particularly 3-deoxyglucosone and methylglyoxal are generated from autoxidation of glucose and the degradation of Amadori products. In the late stage of glycation, irreversible compounds called AGEs are formed through various chemical reactions including direct degradation of Amadori products or Schiff bases, protein modification by dicarbonyl compounds and reactions between Amadori products and AGE precursors. Methylglyoxal (MG) is commonly recognized as the most reactive glycating agent and irreversibly reacts with lysine residues in proteins to form fluorescent crosslinking and non-fluorescent crosslinking AGEs in the last stage of glycation [22, 23]. Our previous findings showed that IFA prevented glucose- and fructose-induced formation of AGE in BSA at the initial stage of glycation resulting in reduced conversion of the initial glycated product to AGEs [18]. In the present study, MG-induced formation of AGEs was also attenuated by IFA at the intermediate stage of glycation. These findings, taken together, suggest that IFA can protect from the initial and intermediate stages of glycation, thus leading to inhibition of the formation of AGEs in the late stage.

Several lines of evidence show that superoxide and hydroxyl radicals can be generated from the reaction between lysine and MG [7]. It has been reported that MG-induced albumin modification generates the cross-linked methylglyoxal dialkylimine radical cation and the enediol radical anion of methylglyoxal during the glycation process [24, 25]. The formation of these intermediates leads to protein cross-linking and formation of radical cation sites on the cross-linked proteins. The presence of trace metal ions (copper and iron ions) enhances hydroxyl radical generation by reacting with hydrogen peroxide (H_2_O_2_) through the Fenton reaction [7]. ROS generated from this reaction contributes oxidative modification of protein and DNA [7]. In the present study, evidence of ROS-induced oxidative modifications included the significant increase of protein carbonyl in BSA as well as DNA damage. In addition, the formation of superoxide anion and hydroxyl radicals generated from lysine and MG was confirmed by the observed increase in reduced cytochrome *c* and TBARS, which was consistent with previous studies [7, 25]. However, when the MG and lysine was incubated with IFA, the increased cytochrome *c* reduction and TBARS level was attenuated suggesting that IFA scavenges ROS. Considerable interest has been devoted to phytochemical compounds due to their ability to prevent lysine/MG-induced protein glycation and DNA damage by acting as free radical scavengers [26] and, our present findings indicate that IFA also acts in this manner. Other mechanisms related to the ability to trap MG have been proposed [27, 28] but these results clearly demonstrated that IFA did not directly react with MG, suggesting that carbonyl scavenging activity is not the antiglycation mechanism of IFA. Further experiments are required to investigate the effect of IFA on MG-induced cell toxicity.

## Conclusion

The results suggest that the mechanism of IFA for the inhibition of MG-induced protein glycation and DNA damage is free radical scavenging of superoxide anion and hydroxyl radical activity without the MG-trapping ability.
